# A Novel, Safe, Non-Adjuvanted Alphavirus RNA Particle Vaccine Expressing the Rabies Virus Glycoprotein Induces a Three-Year Duration of Immunity in Dogs and Cats After a Single Vaccine Dose

**DOI:** 10.3390/vaccines13121176

**Published:** 2025-11-21

**Authors:** Ken Stachura, Randall Davis, Kari Carritt, Mark Mogler, Zach Xu, Ian Tarpey

**Affiliations:** 1 Research and Development Department, Merck Animal Health, Elkhorn, NE 68022, USA; 2 Research and Development Department, Merck Animal Health, Ames, IA 50010, USA; 3 Research and Development Department, MSD Animal Health, 5831 AN Boxmeer, The Netherlands

**Keywords:** rabies, vaccine, glycoprotein, efficacy, safety

## Abstract

Background/Objectives: To this day, rabies remains a significant global threat. This threat remains even with the availability of vaccines for humans, wildlife, and domestic animals, which are used as part of a series of interventions to attempt to control the infection and disease. The number of annual human deaths from rabies globally remains significant, with infections being mainly caused by domestic dogs. Although a number of vaccines exist for domestic animals, most contain inactivated rabies virus with adjuvants. Methods: To investigate alternatives to conventional rabies vaccines for dogs and cats, we developed a novel, non-adjuvanted, low-volume (0.5 mL) vaccine, based on the Venezuelan equine encephalitis virus (VEEV) TC-83-derived RNA particle (RP) expressing the rabies glycoprotein (G). This novel vaccine combines the safety profile of a non-adjuvanted vaccine while inducing consistently high efficacy and an extended duration of immunity similar to that shown by adjuvanted vaccines. Results: In multiple studies, we demonstrated that young kittens and puppies can be safely vaccinated without serious adverse effects. In graded dose experiments with cats and dogs, the RNA particle vaccine induced neutralizing levels of antibodies. Additionally, in vaccination/challenge studies, 100% protection from virulent rabies was demonstrated in excess of three years post-vaccination from a single dose at 12 weeks of age in both dogs and cats. The safety of the RP-Rabies vaccine in dogs and cats as young as twelve weeks of age was demonstrated in field safety studies using two vaccine serials formulated at a field dose. Conclusions: Data from these studies suggest that the RP-Rabies vaccine offers an excellent alternative to current vaccines combining the safety of a non-adjuvanted vaccine in a low-volume, single dose with the induction of an extended duration of immunity of at least three years in both dogs and cats.

## 1. Introduction

Although many tools to control rabies infection are available, it remains a major cause of human mortality, estimated at fifty-nine thousand deaths annually [1]. Rabies is a negative-sense single-stranded RNA virus of the Lyssavirus genus within the Rhabdoviridiae family. The virus is maintained in reservoirs in North America and Europe; wildlife species such as raccoons, foxes and skunks are the typical reservoirs. In Asia, South America, the Middle East, and the Caribbean, the domestic dog is the primary reservoir [2]. Domestic animals, particularly dogs, are responsible for most human rabies infections. To a lesser extent, cats are also responsible for some human infections globally [3,4]. In order to protect domestic animals and reduce human infections, a number of licensed veterinary vaccines are available which have a duration of immunity (DOI) of up to three years [5]. The majority of the available rabies vaccines utilize adjuvants to induce high levels of protection with a long duration of immunity. Studies have demonstrated that vaccines containing adjuvants may induce varying levels of local reactivity in dogs and cats post-vaccination [6,7]. Considering the concern with feline injection site sarcomas and its unknown multifactorial etiology [8], reducing the use of adjuvanted vaccines and the number of injections received is a prudent step in the management of this disease in cats. Rabies vaccines can also contain extraneous proteins from the manufacturing process, including bovine serum albumin (BSA) derived from cell culture media.

The antibody responses developed by the humoral immune system are primarily involved in eliciting protection against rabies, with a virus-neutralizing response of 0.5 international units per milliliter (IU/mL) recognized as an indicator of successful vaccination [9]. It has also been recognized that a cell-mediated response plays a role in rabies immunity [10]. The individual immune response elicited by the available vaccines can vary according to age, sex, size, and breed [11,12], and post-vaccination serological studies demonstrate a variability in terms of immune response [13,14].

The glycoprotein (G) of rabies virus is a well-characterized protective immunogen and is therefore suitable for use in recombinant vector vaccines without requiring adjuvants. Many recombinant vector vaccines have been developed with promising results [15,16,17,18,19] but only one is commercially available utilizing the canarypox virus [20]; this vaccine is only indicated for use in felines. The lack of commercialization of other adjuvant-free vaccines may be associated with the difficulty in achieving a prolonged duration of immunity from a single administration in a primary course.

We developed a safe, low-volume rabies vaccine which provides reliable efficacy with an extended duration of immunity of at least three years with a single vaccination, without relying on an adjuvant for stimulation of the immune response. For this purpose, we utilized alphavirus-based replicon RNA particle technology derived from the attenuated TC-83 strain of the Venezuelan equine encephalitis virus to express the rabies glycoprotein (G). RNA particle technology has been tested in numerous species [21,22] and has been shown to be safe and efficacious in dogs and cats with a variety of antigens [23,24,25]. The RNA particle platform has also been used successfully in multiple swine applications [26,27]. For this vaccine, rabies glycoprotein (G) is inserted in place of the VEEV structural genes. Upon vaccination, the RNA particles, which lack the structural VEEV genes rendering them propagation-defective, deliver the RNA to cells and the self-amplifying RNA that direct the translation of up to 15–20% of the total protein expression in cells [28]. Importantly, RNA particle vaccines have been shown to induce both innate and adaptive immune responses, including virus-neutralizing antibodies and T cell responses [21,23], circumventing the requirement for an adjuvant. Furthermore, RNA particle vaccines do not spread, cannot revert to virulence, and the protein expressed can be used to differentiate infected from vaccinated animals (DIVA) where required.

Via vaccination experiments, we demonstrated that the RNA particle vaccine is safe and that long-lasting antibody responses can be induced with a range of vaccine potencies in 12-week-old puppies and kittens. Furthermore, we demonstrated complete protection in both dogs and cats from rabies challenge for more than three years after a single vaccination with no additional booster dose required to achieve this duration of immunity.

## 2. Materials and Methods

### 2.1. Vaccine Formulations

The open reading frame of rabies virus glycoprotein (G) was codon-optimized and synthesized (ATUM, Newark, CA, USA) with flanking restriction sites to facilitate its cloning into a VEEV TC-83-derived replicon vector. Rabies G-encoding replicon vector DNA and separate VEEV TC-83 capsid and glycoprotein “helper” DNA plasmids were linearized and then in vitro transcribed using RNA polymerase and cap analog (Promega, Madison, WI, USA). Purified RNA for the Rabies G-encoding replicon, capsid, and glycoprotein were combined and co-transfected into Vero cells by electroporation. The helper RNA sequences contain the 5′ and 3′ elements required for trans-amplification by the VEEV replicase but lack the alphavirus subgenomic promoter sequence (known as “promoterless split helpers”) [29]. After the overnight incubation of the cells in serum-free culture media, the replicon RNA particles were purified by depth filtration, eluted in 400 mM NaCl with 5% (*w*/*v*) sucrose buffer, passed through a 0.22 micron filter, and aliquoted prior to downstream formulation and testing. As a control, RNA particles expressing green fluorescent protein (GFP) were also prepared as described above.

The vaccines for the graded dose study were in a liquid formulation and were back-titrated after use to confirm the vaccination dose. The vaccines for the duration of the immunity study were prepared by lyophilization of RNA-particle Rabies or RNA-particle GFP in a stabilizer, stored at 2–8 °C and reconstituted with 0.5 mL sterile water diluent. The titers of functional RNA-particle Rabies or RNA-particle GFP were determined by immunofluorescence assay on infected Vero cell (Alphavax Inc., Research Triangle Park, NC, USA) monolayers. Briefly, the vaccines were serially diluted and added to a Vero cell monolayer culture in 96-well plates and incubated at 37 °C for 18–24 h. After incubation, the cells were fixed and stained with the primary antibody (mouse anti-rabies virus glycoprotein monoclonal antibody) followed by the Alexa Fluor 488-conjugated goat anti-mouse antibody. The plates were read on the BioTek Cytation 5 cell imaging multimode reader with the GFP filter cube using Gen5 Image Prime software version 3.05.11 (Agilent, Santa Clara, CA, USA). RNA particles of each test vaccine were quantified by counting all the positive (fluorescing) cells in each of two wells per dilution. The mean positive cell count for each dilution was multiplied by the dilution factor and divided by the volume inoculated into each well (0.04 mL). In each vaccination study a back-titration of the vaccine used was conducted as described above.

The vaccines for the field safety studies were two commercial serials prepared and lyophilized as described above.

For the graded dose studies, a commercial vaccine containing inactivated whole rabies virus and adjuvanted with aluminum hydroxide (Defensor^®^ 3, Zoetis, Parsippany, NJ, USA) was included. The commercial vaccine, indicated for a three-year duration of immunity in both cats and dogs, was within expiration dating and administered as a 1.0 mL dose according to the manufacturer’s instructions.

### 2.2. Blood Collection

Whole blood samples were collected by venipuncture of the cephalic or saphenous veins using vacutainer needles and evacuated serum separation blood collection tubes (SST). Blood collected into SSTs was allowed to clot at ambient temperature (15–30 °C) and then centrifuged at 2000× *g* for 10 min to separate the serum.

### 2.3. Serology

Neutralizing antibodies against rabies virus in the serum samples were measured using the rapid fluorescent focus inhibition test (RFFIT) [30,31]. The serum samples were heat-inactivated at 56 °C for 30 min, then an equal volume of 25% (*w*/*v*) kaolin was added to the serum samples. The serum samples were diluted five-fold in tissue culture chamber slides. The rabies virus (CVS-11 strain) was diluted and added to each chamber to target between 30 and 100 fluorescing foci dose_50_/chamber. The mixture of serum and virus in chamber slides was incubated at 37 °C for 90 min. BHK-21 cells in media containing 10 µg/mL DEAE-dextran were then added to the serum/virus mixture at 1.6 × 10^5^ cells/chamber and the chamber slides were incubated at 37 °C for 20 to 24 h. The chambers were removed from the slides, and the slides were fixed in cold acetone for 10 min and dried. The fluorophore-conjugated anti-rabies antibody was added to each slide and incubated at 37 °C for 30 min, then rinsed in PBS for 10 min, followed by air-drying. Slides were viewed with a fluorescent microscope at 130×; the number of microscopic fields with at least one cell with fluorescence was recorded. The neutralizing titer was defined as the last dilution of serum in which 10 to 20 microscopic fields contain at least one fluorescing cell. The 50% end-point titer of each serum sample was calculated by the method of Spearman–Karber. A standard reference serum was tested in each assay, sample titers were normalized to the reference serum titer, and the results are reported in international units per milliliter (IU/mL).

### 2.4. Challenge Virus

The New York City street rabies challenge virus was originally isolated from the salivary glands of an infected dog in New York City [32]. Challenge stocks of New York City street rabies were obtained from the Center for Veterinary Biologics, Animal, and Plant Health Inspection Service (Ames, IA, USA). Lot 92-5A (used in the canine three-year DOI study) was passed once in a skunk and three times in foxes; the challenge virus was harvested from the salivary glands. Lot 13–16 (used in the feline three-year DOI study) was prepared from the salivary glands of cats challenged with Lot 92-5A. The virus was diluted in Dulbecco’s Modified Eagles Medium (DMEM) with 2% horse serum and 50 μg/mL gentamicin. The challenge virus was titrated on BHK-21 cells, and the titers were calculated by the Spearman–Karber method (50% endpoint).

### 2.5. Bovine Serum Albumin Assay

The bovine serum albumin (BSA) content of commercial rabies vaccines was quantified with a BSA enzyme-linked immunosorbent assay (ELISA) kit (Cygnus Technologies, Leland, NC, USA). The assay uses two antibodies specific to BSA: a capture antibody coated on microplate strips, and the detection antibody labeled with horseradish peroxidase (HRP). Samples and standards were tested in duplicate. Standards and test samples were incubated in the microplate at 24 °C for one hour on an orbital plate shaker. Following the washing of the test strips, TMB dye substrate was added to each well, and the microplate was incubated statically for thirty minutes. After the addition of the stop solution, the plate was read on a microplate spectrophotometer (SpectraMax^®^ 190, Molecular Devices, San Jose, CA, USA) at a test wavelength of 450 nm and reference wavelength of 650 nm. Optical densities of test samples were compared to the standard curve of BSA using a linear curve fit. The optical density is directly proportional to the concentration of BSA. The commercial rabies vaccines tested were the RNA-particle Rabies vaccine (Nobivac^®^ NXT 3-Rabies, Merck Animal Health, Rahway, NJ, USA), the canarypox-vectored rabies vaccine (Purevax^®^ Feline 3 Year Rabies, Boehringer Ingelheim Animal Health Inc., Athens, GA, USA), and the conventional, inactivated rabies vaccine (Vanguard^®^ Rabies 3 Year, Zoetis, Parsippany, NJ, USA).

### 2.6. Feline Graded Dose Study Design

Purpose-bred, rabies-seronegative cats at 12 weeks of age were blocked by litter and randomly assigned to treatment groups. Ten cats per group were vaccinated subcutaneously with a dose of either 2.7 × 10^7^ (Group 1), 2.6 × 10^6^ (Group 2) or 4.0 × 10^5^ (Group 3) RNA-particle Rabies. An additional five cats were vaccinated subcutaneously with a commercial inactivated, adjuvanted rabies vaccine according to the manufacturer’s instructions (Group 4) (Table 1). Back-titrations of the RNA particle vaccine formulations were conducted to determine the precise doses administered. The cats were observed daily for general health status, and blood was collected to assess serological response at days 30, 58, 91, 120, 149, 182, 268, and 359. All personnel testing the laboratory samples were blinded to the treatment groups.

### 2.7. Canine Graded Dose Study Design

Purpose-bred, rabies-seronegative dogs at 12 weeks of age were blocked by litter and randomly assigned to treatment groups. Five dogs per group were vaccinated subcutaneously with a dose of either 4.0 × 10^8^ (Group 1), 5.0 × 10^7^ (Group 2) or 8.3 × 10^6^ (Group 3) RNA-particle Rabies vaccine. An additional five dogs were vaccinated subcutaneously with a 1 mL dose of a commercial, inactivated, adjuvanted rabies vaccine according to the manufacturer’s instructions (Group 4). A fifth treatment group of five dogs was vaccinated subcutaneously with a placebo RNA particle vaccine expressing a protein from an unrelated virus (Group 5) (Table 2). Back-titrations of the RNA particle vaccine formulations were conducted to determine the precise doses administered. The dogs were observed daily throughout the course of the study and bled to assess serological responses at days 30, 59, and 90. All personnel testing the laboratory samples were blinded to the treatment groups.

### 2.8. Feline Three-Year Duration of Immunity Study Design

Purpose-bred, rabies-seronegative cats at 12 weeks of age were blocked by litter and randomly assigned to treatment groups. A group of 30 cats was vaccinated subcutaneously with a 0.5 mL dose of RNA-particle Rabies vaccine. A second group of 15 cats was vaccinated subcutaneously with a 0.5 mL dose of RNA-particle GFP as a placebo. Both groups were housed together in a single room throughout the study. The cats were observed daily for general health status throughout the course of the study. Blood was collected to assess serological response at days 0, 30, 90, 181, 269, 365, 545, 731, 916, and 1100. During the three-year holding period, prior to challenge, two cats in the RNA-particle Rabies vaccinated group and one cat in the placebo group were removed from the study for health reasons not associated with the vaccines. The remaining 28 cats in the RNA-particle-Rabies-vaccinated group and 14 cats in the placebo group were challenged intramuscularly with the rabies virus at day 1100 and observed for 90 days for clinical signs of rabies. Cats exhibiting clinical signs compatible with rabies were humanely euthanized and considered as deaths due to rabies. The presence of the rabies virus was confirmed in each euthanized cat by the direct fluorescent antibody test. All personnel performing clinical observations and testing the laboratory samples were blinded to the treatment groups.

### 2.9. Canine Three-Year Duration of Immunity Study Design

Purpose-bred, rabies-seronegative dogs at 12 weeks of age were blocked by litter and randomly assigned to treatment groups. A group of 29 dogs was vaccinated subcutaneously with a 0.5 mL dose of RNA-particle Rabies vaccine. A second group of 13 dogs was vaccinated subcutaneously with a 0.5 mL dose of RNA-particle GFP as a placebo. All animals were housed together in a single room throughout the study. The dogs were observed daily for general health status throughout the study. Blood was collected to assess serological response at days −1, 28, 90, 180, 271, 365, 545, 728, 916, 1093, and 1105. During the three-year holding period, prior to challenge, two dogs in the RNA-particle Rabies vaccinated group were removed from the study for health reasons unrelated to the test vaccine. The remaining 27 dogs in the RNA-particle-Rabies-vaccinated group and 13 dogs in the placebo group were challenged intramuscularly with rabies virus on study day 1106 and observed for 90 days for clinical signs of rabies. Dogs exhibiting clinical signs compatible with rabies were humanely euthanized and considered as deaths due to rabies. The presence of the rabies virus was confirmed in each euthanized dog by the direct fluorescent antibody test. All personnel performing clinical observations and testing the laboratory samples were blinded to the treatment groups.

### 2.10. Feline Field Safety Study Design

A total of 665 cats were vaccinated with one of two commercial serials of RNA-particle Rabies vaccine, formulated at a typical field dose in 0.5 mL. The cats were a mixture of purpose-bred and client-owned cats from ten different sites in eight states in the USA representing many breeds. In total, 200 cats were twelve weeks of age, 64 cats were between thirteen weeks and one year of age, and 401 cats were between one and twenty one years of age. Cats were observed at the time of vaccination and each day for 14 days following vaccination for any adverse events.

### 2.11. Canine Field Safety Study Design

A total of 622 dogs were vaccinated with one of two commercial serials of RNA-particle Rabies vaccine, formulated at a typical field dose in 0.5 mL. The test animals included both purpose-bred and client-owned dogs belonging to multiple breeds, from seven different sites in seven states in the USA. The age of dogs enrolled in the study ranged from twelve weeks of age (206 dogs), between thirteen weeks and one year of age (21 dogs), and between one year and sixteen years of age (395 dogs). Dogs were observed at the time of vaccination and each day for 14 days following vaccination for any adverse events.

## 3. Results

### 3.1. Feline Graded Dose Study

**Figure 1:**
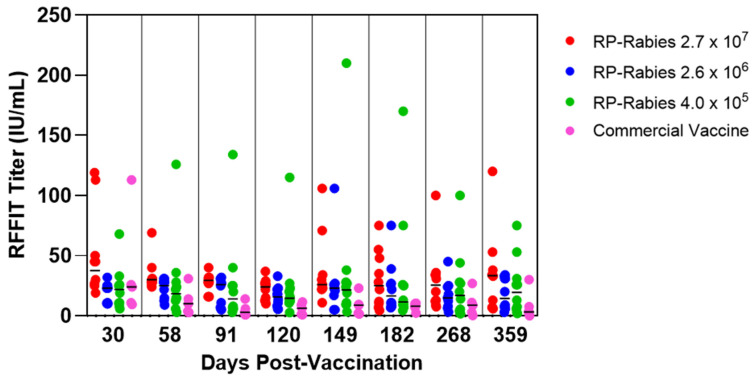
Feline graded dose study rabies-neutralizing antibody response measured by RFFIT. Bar is median group titer.

The RP-Rabies vaccine induced neutralizing antibodies (≥0.5 IU/mL) in all 30 vaccinated cats in the study irrespective of dose (Figure 1). The median serum RFFIT antibody responses from the cats vaccinated with the RP-Rabies vaccines (Groups 1, 2, and 3) were 37.5 (range of 19.0 to 119.0), 23.0 (range of 10.0 to 32.0), and 22.0 (range of 6.0 to 68.0), respectively, at 30 days post-vaccination. At 30 days post-vaccination the commercial adjuvanted vaccine induced a median response of 24.0 IU/mL (range of 9.4 to 113.0 IU/mL). However, at 58 days post-vaccination the median titer in the commercial vaccine group had dropped to 10.0 IU/mL (range 2.8 to 31.0 IU/mL) while all three RP-Rabies-vaccinated groups’ median titers were at 18.4 IU/mL or above (range 3.1 to 126.0 IU/mL). The RP-Rabies-vaccinated groups maintained these levels throughout the remainder of the study and at no point did any single animal’s titer drop below 0.5 IU/mL. In contrast, at the last two time points tested (days 268 and 359), the titer of one cat in the commercial vaccine group dropped below the 0.5 IU/mL level.

### 3.2. Canine Graded Dose Study

**Figure 2:**
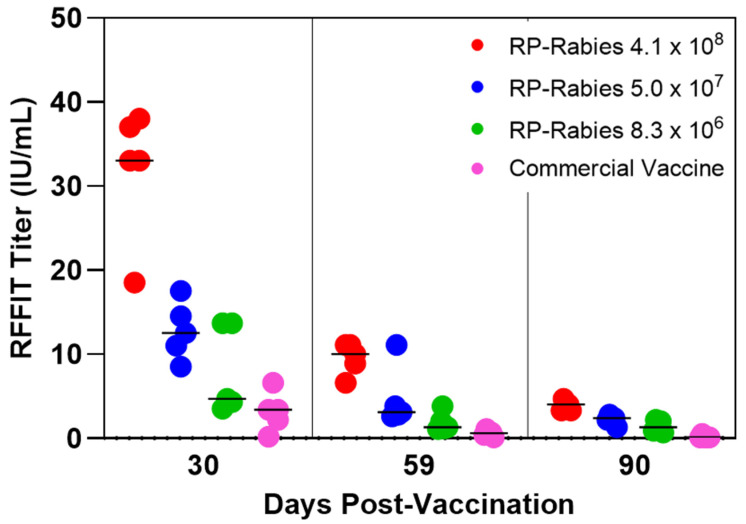
Canine graded dose study of rabies-neutralizing antibody response measured by RFFIT. Bar is median group titer.

The RP-Rabies vaccine induced neutralizing antibodies (≥0.5 IU/mL) in all 15 vaccinated dogs in the study irrespective of the dose (Figure 2). At 30 days post-vaccination, the commercial adjuvanted vaccine induced a median response of 3.4 IU/mL (range of 0.2–6.6). At day 30, the median serum RFFIT antibody responses for the dogs vaccinated with the RP-Rabies vaccines (Groups 1, 2, and 3) were 33.0 IU/mL (range of 18.5–38.0), 12.5 IU/mL (range of 8.5–17.5), and 4.7 IU/mL (range of 3.5–13.7), demonstrating a dose titration effect. At day 59, the median titer for dogs receiving the commercial vaccine had dropped to 0.6 IU/mL (range of <0.1–1.1), while among the three RP-Rabies-vaccinated groups, median RFFIT titers were 10.0 IU/mL (range of 6.6–11.1) for Group 1, 3.1 IU/mL (range of 2.6–11.1) for Group 2, and 1.3 IU/mL (range of 1.1–3.8) for Group 3. At day 90, the median titer for dogs receiving the commercial vaccine had dropped further to 0.1 IU/mL (range of <0.1–0.5) while the RP-Rabies-vaccinated groups had median titers of 4.0, 2.4, and 1.3 IU/mL for Groups 1–3, respectively. All RNA-particle-Rabies-vaccinated dogs (Groups 1, 2, and 3) maintained RFFIT titers of 0.5 IU/mL or greater through day 90 post-vaccination. In contrast, in the commercial vaccine group one dog never reached a titer of 0.5 IU/mL and four out of five dogs’ titers had dropped below 0.5 IU/mL by day 90.

### 3.3. Feline Three-Year Duration of Immunity Study

**Figure 3:**
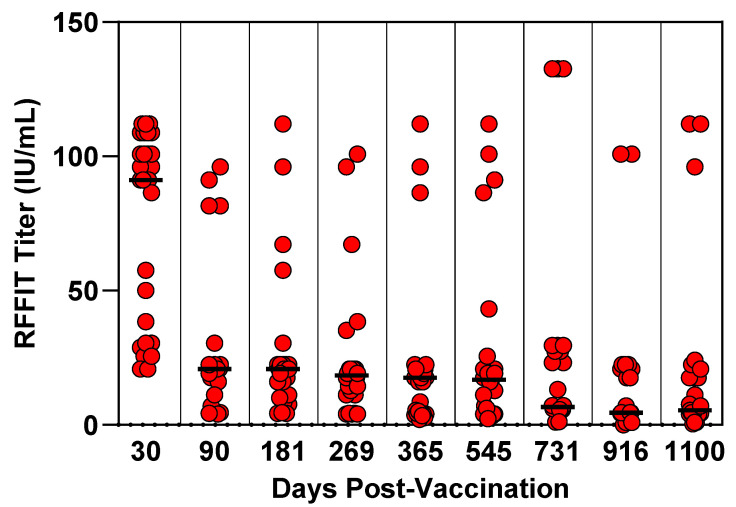
Feline three-year duration of immunity study. Blood samples were collected at intervals and tested for rabies-neutralizing antibody response by RFFIT. Only the vaccinate group is shown. Bar is median group titer.

Prior to vaccination, all cats had rabies antibody RFFIT titers ≤ 0.2 IU/mL, indicating the cats were seronegative at the time of vaccination. The RP-Rabies vaccine induced neutralizing antibodies (≥0.5 IU/mL) in all cats that were still enrolled at the end of the study, with a peak median value at day 30 of 91.2 IU/mL (range 20.8 to ≥112.0 IU/mL) and of 5.4 IU/mL on the day of challenge, day 1100 (range 0.4 to ≥112.0 IU/mL) (Figure 3). The placebo group did not seroconvert to rabies at any time during the study. Following challenge with virulent rabies, all 14 of the placebo-vaccinated control cats developed clinical signs compatible with rabies and were euthanized. Rabies virus infection was confirmed in all 14 control cats by direct fluorescent antibody testing of brain tissue. All 28 RP-Rabies-vaccinated and challenged cats remained in good health throughout the monitoring period.

### 3.4. Canine Three-Year Duration of Immunity Study

**Figure 4:**
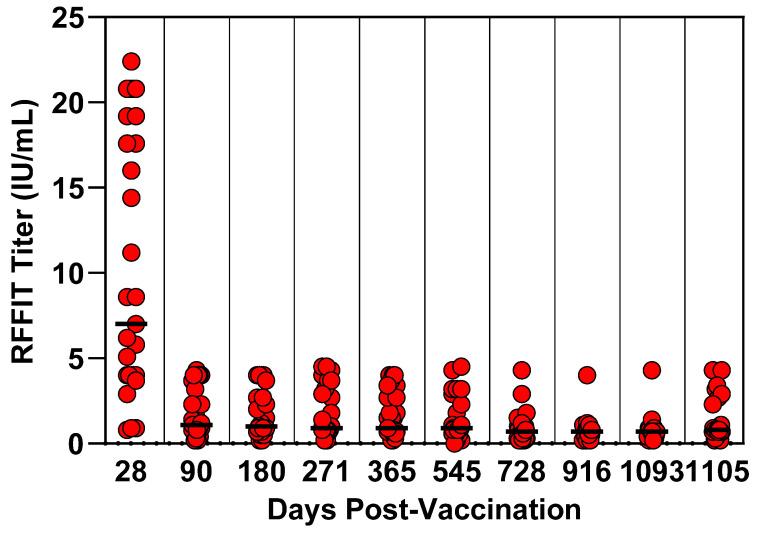
Canine three-year duration of immunity study. Blood samples were collected at intervals and tested for rabies-neutralizing antibody response by RFFIT. Only the vaccinate group is shown. Bar is median group titer.

Prior to vaccination, all dogs had rabies antibody RFFIT titers ≤ 0.2 IU/mL, indicating the dogs were seronegative at the time of vaccination. The RP-Rabies vaccine induced neutralizing antibodies in all dogs with a median titer at day 28 of 7.0 IU/mL (range 0.8 to 22.4 IU/mL) and of 0.8 IU/mL on the day of challenge, day 1105 (range 0.2 to 4.3 IU/mL) (Figure 4). The placebo group did not seroconvert to rabies at any time during the study. Following challenge with virulent rabies, 11 out of 13 (85%) of the placebo-vaccinated control dogs developed clinical signs compatible with rabies and were euthanized. Rabies virus infection was confirmed in 100% (11/11) of these controls by direct fluorescent antibody testing of brain tissue. All 27 dogs that received the RP-Rabies vaccine remained in good health throughout the post-challenge monitoring period.

### 3.5. Feline Field Safety Study

Among the 665 vaccinated cats there were 38 cats (5.7%) that experienced an adverse event deemed related to the vaccine. The most commonly reported adverse events were injection site pruritis (2.1%) and lethargy (1.4%). Only two cats were reported with injection site swelling and both of these resolved within one day without treatment. The data supports the finding that the RNA-particle Rabies vaccine is safe for use in cats as young as 12 weeks of age.

### 3.6. Canine Field Safety Study

Among the 622 vaccinated dogs there were 42 dogs (6.8%) that experienced an adverse event deemed related to the vaccine. The most commonly reported adverse events related to the vaccine were lethargy (2.6%), loss of appetite (1.3%), and vomiting (1.1%). Only two dogs were reported with injection site swelling and both of these resolved within two days without treatment. The data supports the finding that the RNA-particle Rabies vaccine is safe for use in dogs as young as 12 weeks of age.

### 3.7. Bovine Serum Albumin Content

**Figure 5:**
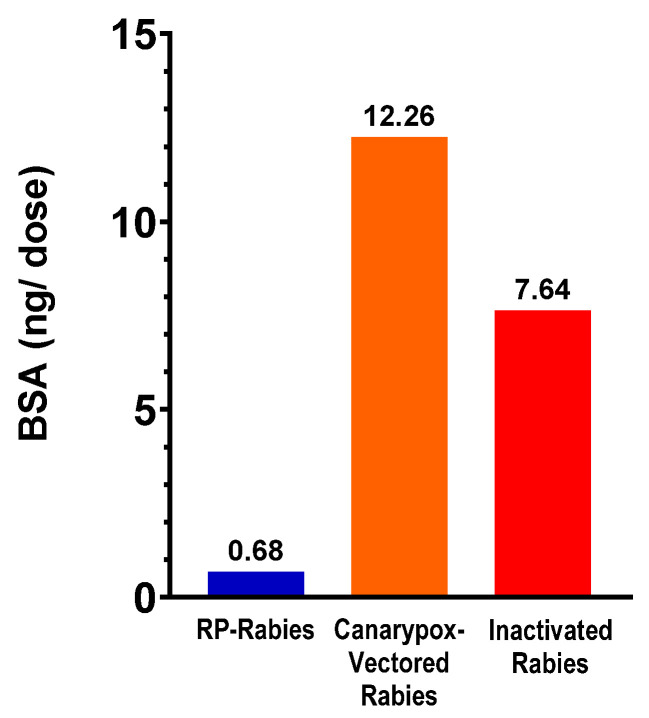
Bovine serum albumin content of commercial rabies vaccines as determined by BSA ELISA.

Three commercial rabies vaccines were tested for bovine serum albumin content. The canarypox-vectored rabies vaccine contained 18 times more BSA compared to the RP-Rabies vaccine, and the conventional, inactivated rabies vaccine contained 11 times the BSA compared to the RP-Rabies vaccine (Figure 5).

## 4. Discussion

Rabies is a communicable disease spread between infected wildlife, domestic animals, and to humans via bites and/or saliva. Given the lethal outcome from infection, vaccination remains the primary defense against disease. While several rabies vaccines for domestic animals are available, they differ in their design characteristics (such as whether or not they contain an adjuvant), dose volume, and in their ability to induce consistent, long-lasting antibody responses [14]. Adjuvanted vaccines are typically formulated with the whole, inactivated rabies virus and aluminum-based adjuvants, and are formulated to induce an inflammatory response at the point of vaccination, which stimulates the host immune response. Adjuvanted vaccines have been associated with an increased occurrence of local reactions at the site of inoculation and there has been debate over many years as to whether they are one of the many factors contributing to the development of feline injection site sarcoma [7,8]. The formulation of rabies vaccines can also impact safety; vaccines can contain residual proteins from culture media including BSA, which has been implicated in hypersensitivity reactions in dogs [33] and cats [34].

Given the concern around the number of vaccinations given to dogs and cats, especially those containing adjuvants, we developed a low-volume (0.5 mL), non-adjuvanted vaccine which is administered as a single 0.5 mL dose at 12 weeks of age to develop a three-year duration of immunity. We used an RNA particle based on the VEEV strain TC-83, which is used to deliver replicon RNA to the cells for the expression of the rabies glycoprotein (G). By inducing a robust innate and adaptive immune response, the RNA particle platform offers long-lasting immunity from rabies with a single vaccination. The manufacturing process of the RNA particle does not require antimicrobials or preservatives, and RNA particles are purified during the harvesting step, reducing residual cell culture components such as BSA and other serum proteins in the final product. The RNA-particle Rabies vaccine had 94% less BSA than a canarypox-vectored rabies vaccine and 91% less BSA than a conventional, inactivated rabies vaccine. The production of the RNA-particle Rabies vaccine requires specialized manufacturing equipment and technology and because the final product is in lyophilized form and the batch size is limited by the capacity of lyophilization equipment. The production cost of the vaccine is higher than a typical inactivated rabies vaccine. The finished product is stable for three years when stored at 2–8 °C.

In the experiments, it was demonstrated that the RP-Rabies vaccine was able to induce protective levels of rabies-neutralizing antibodies in cats and dogs from a single dose. A graded dose study in cats demonstrated that a vaccine dose as low as 4 × 10^5^ RP (approximately 250 times lower than the dose of the RP-Rabies commercial vaccine) could induce a protective level of antibody which persisted in cats for over one year. In contrast, one of the five cats vaccinated with the inactivated, adjuvanted vaccine had an antibody response that dropped below 0.5 IU/mL at day 268 of the study, a finding in line with previous studies showing variable responses with inactivated vaccines in cats [14]. In a graded dose study in dogs, a vaccine dose as low as 8.3 × 10^6^ RP induced a protective RFFIT titer of at least 0.5 IU/mL in all vaccinated dogs that persisted to 90 days post-vaccination. In contrast, the commercial, inactivated vaccine induced a titer of at least 0.5 IU/mL in only four out of five vaccinated dogs. In the feline three-year duration of immunity study, the RP-Rabies vaccine formulated at the minimum protective dose was able to consistently induce long-lasting antibody responses, with all animals maintaining seropositivity for over three years and all vaccinates fully protected against virulent rabies challenges without an interim booster. In the canine three-year duration of immunity study, all 27 dogs vaccinated with an RP-Rabies vaccine at the minimum protective dose were fully protected against virulent rabies challenge without an interim booster. In the field safety studies in dogs and cats, the RP-Rabies vaccine formulated at a field dose was safe and well-tolerated by cats and dogs of various breeds and ages, including 12-week-old animals. The results further demonstrate the broad potential use of the RNA particle based on VEEV [21,23,24,25,26,27].

## 5. Conclusions

Overall, this data demonstrates that the rabies vaccine based on replicon RNA particles is safe and provides high-level, long-lasting protection with a single dose in cats and dogs. The RNA-particle Rabies vaccine, licensed under the tradename Nobivac^®^ NXT 3-Rabies, is labeled for use in dogs and cats as young as 12 weeks of age, has established a duration of immunity of at least 3 years [35], and will be a valuable asset in the ongoing control of rabies in domestic animals.

## Figures and Tables

**Table 1:** Feline graded dose study design.

Treatment Group	Number of Cats	Vaccine
1	10	RNA-particle Rabies 2.7 × 10^7^ RNA particles/dose
2	10	RNA-particle Rabies 2.6 × 10^6^ RNA particles/dose
3	10	RNA-particle Rabies 4.0 × 10^5^ RNA particles/dose
4	5	Commercial Rabies Vaccine

Vaccine potencies are based on back-titration of vaccines.

**Table 2:** Canine graded dose study design.

Treatment Group	Number of Dogs	Vaccine
1	5	RNA-particle Rabies 4.0 × 10^8^ RNA particles/dose
2	5	RNA-particle Rabies 5.0 × 10^7^ RNA particles/dose
3	5	RNA-particle Rabies 8.3 × 10^6^ RNA particles/dose
4	5	Commercial Rabies Vaccine
5	5	Placebo RNA particle Vaccine

Vaccine potencies are based on back-titration of vaccines.

## Data Availability

The original contributions presented in this study are included in the article. Further inquiries can be directed to the corresponding author.
